# How Sequential Interactive Processing Within Frontostriatal Loops Supports a Continuum of Habitual to Controlled Processing

**DOI:** 10.3389/fpsyg.2020.00380

**Published:** 2020-03-10

**Authors:** Randall C. O’Reilly, Ananta Nair, Jacob L. Russin, Seth A. Herd

**Affiliations:** ^1^Computational Cognitive Neuroscience Lab, Department of Psychology, Computer Science, and Center for Neuroscience, University of California, Davis, Davis, CA, United States; ^2^eCortex, Inc., Boulder, CO, United States

**Keywords:** habits, goals, controlled processing, automatic processing, computational modeling, frontal cortex, basal ganglia

## Abstract

We address the distinction between habitual/automatic vs. goal-directed/controlled behavior, from the perspective of a computational model of the frontostriatal loops. The model exhibits a continuum of behavior between these poles, as a function of the interactive dynamics among different functionally-specialized brain areas, operating iteratively over multiple sequential steps, and having multiple nested loops of similar decision making circuits. This framework blurs the lines between these traditional distinctions in many ways. For example, although habitual actions have traditionally been considered purely automatic, the outer loop must first decide to allow such habitual actions to proceed. Furthermore, because the part of the brain that generates proposed action plans is common across habitual and controlled/goal-directed behavior, the key differences are instead in how many iterations of sequential decision-making are taken, and to what extent various forms of predictive (model-based) processes are engaged. At the core of every iterative step in our model, the basal ganglia provides a “model-free” dopamine-trained Go/NoGo evaluation of the entire distributed plan/goal/evaluation/prediction state. This evaluation serves as the fulcrum of serializing otherwise parallel neural processing. Goal-based inputs to the nominally model-free basal ganglia system are among several ways in which the popular model-based vs. model-free framework may not capture the most behaviorally and neurally relevant distinctions in this area.

## Introduction

Since its inception, the field of psychology has been fascinated by the distinction between two types of behavior, one that leads us to act relatively automatically, according to well-worn habits, and another that allows us to act with intent and deliberation (James, 1890; Thorndike, 1911; Hull, 1943; Tolman, 1948). These two classes of thought and action have been referred to by several different sets of terminologies, each with slightly varying definitions, which has sown some confusion in the literature (Hassin et al., 2009; Kool et al., 2018; Miller et al., 2018, 2019). Historically, the first terminology applied to this intuitive distinction was *stimulus-response* vs. *cognitive-map guided* (Thorndike, 1911; Tolman, 1948). This distinction was later replaced by *habitual* vs. *goal-directed behavior* (Tolman, 1948; Balleine and Dickinson, 1998; Dickinson and Balleine, 2002; Killcross and Coutureau, 2003; Balleine, 2005; Yin and Knowlton, 2006; Tricomi et al., 2009), which co-existed alongside *automatic* vs. *controlled* processing (Shiffrin and Schneider, 1977; Cohen et al., 1990; Miller and Cohen, 2001). More recently, a good deal of work has been directed at the distinction between *model-free* and *model-based* reinforcement learning (Sutton and Barto, 1998; Doya, 1999; Doya et al., 2002; Daw et al., 2005).

In this paper, we attempt to clarify the relationships among these terminological distinctions through the lens of a computational model of the underlying brain mechanisms. This model builds on detailed neural recording data available on animal action-selection. One of the major conclusions from this model is that these apparently distinct types of behavior may be manifestations of a core underlying neural system, which evaluates the relative cost/benefit tradeoffs of engaging in more time-consuming, deliberative processing using the same basic mechanisms that drive all the other behavioral decisions that an organism must make. Furthermore, we argue that the neural pathways that support the habitual stimulus-response level behavior are actually an integral part of the same system that supports deliberative, controlled processing. Thus, this framework provides a unified view of action selection and decision making from the most basic habitual level up to the most complex, difficult decisions that people face. In our theory, Type 2 (deliberative) decisions are essentially composed of many Type 1 (automatic) decisions. Thus, it offers a mechanistic explanation of the proposed continuum between them (Melnikoff and Bargh, 2018).

### Goal-Driven/Controlled vs. Habitual/Automatic

We first establish some common ground by attempting to define a consensus view about the closely-related distinctions between *goal-driven* vs. *habitual*, and *controlled* vs. *automatic* processing. Of the two, controlled vs. automatic (Shiffrin and Schneider, 1977) is perhaps more clearly defined, by virtue of a history of computational models based on the idea that the prefrontal cortex (PFC) supports controlled processing by maintaining active working memory representations that drive top–down biasing of processing elsewhere in the brain (Cohen et al., 1990; Miller and Cohen, 2001; Herd et al., 2006; O’Reilly, 2006; O’Reilly and Frank, 2006). Cognitive control is needed to support novel, difficult, complex tasks, e.g., to overcome prepotent (i.e., habitual) response pathways in the widely-studied Stroop task. As a task or stimulus-response pathway becomes more strongly practiced, behavior becomes more automatic and free from the need for this top–down biasing support. Thus, automatic and habitual are closely related terms. The connection between goal-driven and controlled processing is somewhat less exact, as one could imagine behaving according to goals that do not require significant cognitive control (Bargh, 1989), and potentially even exerting cognitive control in the absence of clear goal-driven motivations. Sustained active neural firing of goal-like representations, that can exert an ongoing biasing effect on behavior, is perhaps a more direct mechanistic connection between the two.

Phenomenologically, habitual behavior is typically characterized as being relatively insensitive to the current reward value of actions, and not as strongly under the control of active, conscious goal engagement (Wood and Rünger, 2016). On the other hand, it remains a challenge to consider the nature of real-world behaviors that are characterized as habits, as they often involve extended sequences of actions coordinated over reasonably long periods of time (e.g., driving home from work, making coffee, etc.) – these do not seem to be entirely unconscious activities devoid of any cognitive control influences, or contextual sensitivity (Cushman and Morris, 2015). Furthermore, how can it be that subtle, unconscious factors can sometimes strongly shape our overt behavior (Bargh, 2006; Huang and Bargh, 2014)?

Our general answer to these questions, as captured in our computational modeling framework, is that both habits and more controlled, goal-driven behaviors emerge from a shared neural system, and both operate within a common *outer-loop* of overall cognitive control that pervasively shapes and modulates the nature of processing performed in the *inner-loops* associated with specific task performance. This is similar to the hierarchical control framework of Cushman and Morris (2015), except that we postulate a sequential, temporal organization of decision making and control, where the same neural systems iteratively process multiple steps over time, including periodic revisiting of the broader context and goals that we refer to as the *outer-loop*. Thus, habits only drive behavior when permitted by this outer-loop of cognitive control, and indeed the actual unfolding of behavior over time is usually at least somewhat coordinated by the outer-loop. Furthermore, as we’ll elaborate below, a crucial factor across all behavior in our framework is a so-called *Proposer* system that integrates many different factors in a *parallel-constraint-satisfaction* system to derive a proposed plan of action at any point in time – the properties of this system may explain how unconscious factors can come to influence overt behavior in the course of solving the *reduction problem* of choosing one plan among many alternatives (Bargh, 2006; Huang and Bargh, 2014).

### The Model-Free vs. Model-Based Dichotomy

Within the above context, how does the *model-based* vs. *model-free* (MBMF) framework fit in? This framework has engaged new enthusiasm by offering the promise of a more formal, precise definition of the relevant processes, and by leveraging the direct connection between reinforcement learning principles and properties of the midbrain dopamine system (Montague et al., 1996; Schultz, 2013). Specifically, the model-free component is typically defined as relying on learned, compiled estimates of future reward associated with a given current state (or potential actions to be taken in that state), which have been trained via phasic dopamine-like temporal difference signals, as in the classic TD and Q-learning reinforcement learning frameworks (Sutton and Barto, 1998). By contrast, the model-based system adds an internal model that can simulate the evolution of the state of the world over multiple iterations, so that action selection can be based on those predicted states. As such, the model-free system is considered to be relatively inflexible to changes in the reward function, including changes resulting from internal state (e.g., not being hungry at the moment), whereas the model-based system can dynamically adjust its predictions based on goal changes and other changes, and is thus more flexible.

Thus, it is this key difference in the relative flexibility of the two systems that maps onto the existing notions of goal-driven vs. habitual behavior. However, there are various other aspects of the MBMF framework which map less well, creating significant confusion when people intend to characterize the goal-driven vs. habitual distinction, but using the MBMF terminology. At a very basic level, there is no principled reason why a model-free system should not have access, as inputs, to internal drive and goal states in addition to external environmental states. If it does, its behavior can also be goal-directed, and sensitive to internal bodily states such as hunger. In addition, model-based is not synonymous with goal-directed, as model-based is defined specifically in terms of models of the external environment. In our framework, a model-free-like system indeed receives internal state and goal inputs, and thus participates in goal-directed behavior. This illustrates an important mismatch between these two terminologies, which are often taken to be interchangeable. More generally, standard reinforcement-learning paradigms tend not to incorporate a significant goal-driven component, and instead generally assume a single overriding goal of maximizing a scalar-valued reward, which is delivered to an entirely externally-motivated agent (O’Reilly et al., 2014). Thus, aside from a few more recent examples (Berseth et al., 2018), standard reinforcement-learning models are not particularly well-suited for describing goal-driven processing in the first place.

Recent reviews by Miller et al. (2018, 2019) point out the following additional issues with the MBMF terminology. First, it is problematic that the model-free system relies on learned *value* estimates to drive action selection, whereas most existing data indicates that habitual behavior is specifically more insensitive to reward value (Wood and Rünger, 2016). Second, the neural substrates associated with MBMF mechanisms are largely overlapping and hard to disentangle, involving the dopaminergic system, the basal ganglia (BG), and the prefrontal cortex (PFC). Whereas the BG was traditionally viewed as being primarily a habit-based motor area (e.g., Miller, 1981; Mishkin et al., 1984; Squire and Zola-Morgan, 1991; Squire, 1992; Packard and Knowlton, 2002) more recent evidence and theorizing suggest that, with the exception of the dorsal-lateral striatum, most of the BG is more clearly involved with non-habitual behavior and deliberative, controlled cognition in novel and challenging tasks (Pasupathy and Miller, 2005; Samejima et al., 2005; Yin et al., 2005; Balleine et al., 2007; Seger and Spiering, 2011; Pauli et al., 2012). Many authors nevertheless continue to assume the simple association of model-free with the BG, in keeping with the traditional habit-based ideas.

Furthermore, while the MBMF distinction is often considered to be dichotomous, more recent work has explored various combinations of these aspects to deal with the computational intractability of full model-based control, further blurring the lines between them (Pezzulo et al., 2013; Cushman and Morris, 2015). Likewise, there are many ways of approximating aspects of model-based predictions of future outcomes that may not fit the formal definition of iterative model-state updating, e.g., using predictive learning in the *successor-representation* framework (Dayan, 1993; Littman and Sutton, 2002; Momennejad et al., 2017; Russek et al., 2017; Gershman, 2018). This may be considered acceptable if the distinction is just that the model-free system has absolutely no model-like element, and the model-based system has any kind of approximation of a world model (Daw and Dayan, 2014), but this may end up straining the value of the distinction. For instance, a successor-representation model is otherwise quite similar to a standard model-free system, but it does use information about outcomes (although they do not usually explicitly predict an outcome).

The above considerations led Miller et al. (2018, 2019) to conclude that MBMF are both aspects of the goal-based, controlled-processing system, based on the prefrontal cortex/basal ganglia/dopamine circuits in the brain, while habitual, automatic processing is supported by an entirely separate system governed by a Hebbian, associative form of learning that strengthens with repetition.

### Overview of the Paper

In the remainder of this paper, we present an alternative framework based on computational models of the basal ganglia/prefrontal cortex/dopamine system, which is consistent with the overall critique of MBMF by Miller et al. (2018, 2019), and provides a specific set of ways in which these brain systems can support a continuum of goal-directed, model-based forms of decision making and action selection. The original controlled vs. automatic distinction has always incorporated this notion that these are two poles along a continuum. Our framework goes further in describing how model-based and model-free elements interact in various ways and to varying degrees to provide a rich and multi-dimensional continuum of controlled, goal-driven cognition, which also supports varying degrees and shades of habitual or automatic elements.

This framework contrasts with several others that posit strongly dichotomous and internally homogenous habitual vs. goal-driven pathways, followed by an arbiter system that decides between the two (e.g., Daw et al., 2005; Miller et al., 2019). Instead, we propose that an outer-loop of goal-driven, but model-free, processing is itself essentially an arbiter of how much time and effort to invest in any given decision-making process. It controls the degree of engagement of a broader toolkit of basic decision-making computations to be deployed, as a function of their relative tradeoffs (c.f., Pezzulo et al., 2013). In particular, it controls whether to perform additional steps of predictive modeling down each given branch of the state-space model.

We also address a critical phenomenon for any model in this domain, which is the nature of the transition from controlled to automatic processing (Cohen et al., 1990; Gray et al., 1997; Hikosaka and Isoda, 2010). Behaviorally, this transition occurs gradually over time and appears to reflect something like the strengthening of habit representations, which offer advantages in terms of speed, resistance to distraction, and the ability to do more in parallel, at the cost of flexibility and sensitivity to current goals – i.e., the fundamental underlying tradeoffs along this dimension. However, due to the multi-component nature of our goal-driven model, there are also various ways in which learning within this system can change these relative tradeoffs, leading to a richer picture of this process of habit formation.

## The Proposer-Predictor-Actor-Critic Model

Our theoretical framework has been specified as a neural network model in the Leabra framework (O’Reilly, 1998; O’Reilly and Munakata, 2000; O’Reilly et al., 2016).

**FIGURE 1:**
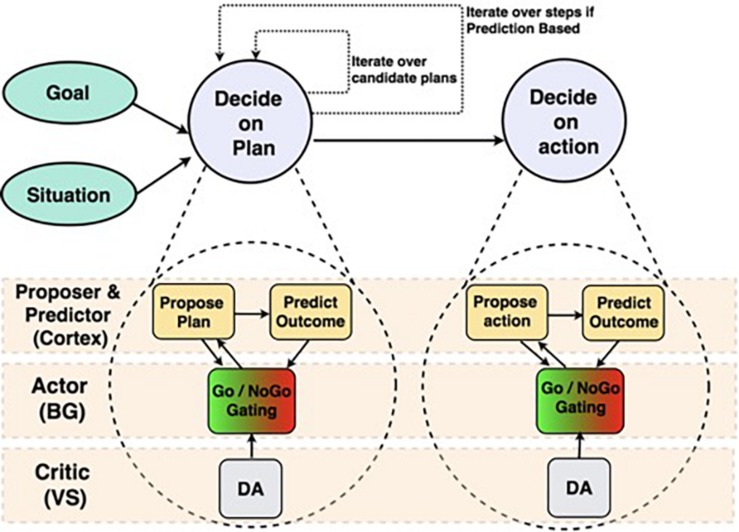
Structure of Proposer-Predictor-Actor-Critic architecture (Herd et al., 2019) across frontal cortex and subcortical areas. We depict two parallel circuits with a hierarchical relationship. The top is a broad functional diagram, emphasizing the serially iterative and hierarchical nature of our proposed decision-making process. The bottom expands those functions, and identifies the brain areas that perform each function.

**FIGURE 2:**
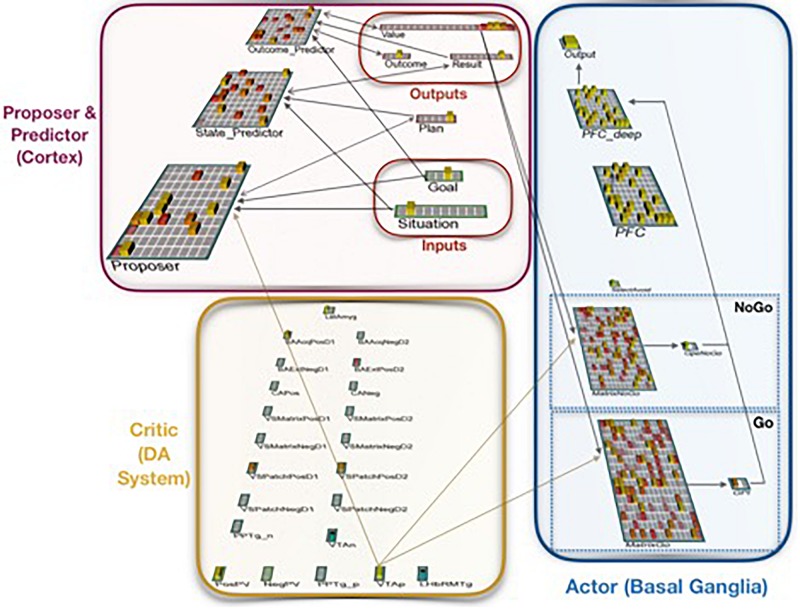
Neural network implementation of the Proposer-Predictor-Actor-Critic theory. The model performs a three-factor task of choosing a Plan that accomplishes a current Goal in a current Situation. This abstract task can be conceptualized as navigation, social interaction, etc. The network’s Proposer component selects one Plan, based on pattern completion from inputs representing the current Situation and the current Goal. Each Plan deterministically produces an Outcome, each of which has one associated Result. The model is rewarded if that Result matches the current Goal. The Predictor component (when it is used) then predicts the resulting Outcome and Result (based on the proposed Plan and the current Situation), and the Actor component then uses that prediction as input to accept or reject that plan. If the plan is rejected, this computational cycle begins again with a new plan from the Proposer.

The *Proposer-Predictor-Actor-Critic* (PPAC) model (Figures 1, 2; Herd et al., 2019) leverages the prototypical loops descending from all areas of frontal cortex through the basal ganglia and converging back to modulate the function of matching areas of frontal cortex (Alexander et al., 1986; Haber, 2010, 2017; Sallet et al., 2013). Functionally, these BG/PFC loops support the ability to selectively activate and maintain neural activity (i.e., working memory) in the service of supporting top-down control representations (Miller and Cohen, 2001; Frank and O’Reilly, 2006; Herd et al., 2006; O’Reilly, 2006). As such, this system is critical for controlled, goal-driven processing. The PPAC model includes an important distinction among the nature of the cortical input representations into the BG: proposed actions vs. predicted outcomes. Critically, complex decision-making unfolds sequentially across multiple iterations in the model, each of which involves parallel operations across these circuits (i.e., a *serial-parallel* model, in which parallel computations are iterated serially).

In this theory, complex decision-making consists of a series of selections of internal “actions,” each of which consists of an update to working memory and/or episodic memory. Selecting a move in chess or choosing a plane ticket to purchase may each require a large number of belief updates (like “too expensive to fly direct in the afternoon”) and the selection of several new mid-level plans (like “try to threaten a more valuable piece instead of defending the knight”). Each of these can be stored in active memory, which executes controlled processing (by exerting top-down biasing of processing (Cohen et al., 1990; Miller and Cohen, 2001; Herd et al., 2006). Maintaining each plan or belief in working memory can also create an episodic memory trace for later recall and re-use. Our theory holds that each such representation is selected for maintenance (and therefore plan execution) much as motor representations are selected, by distinct but computationally and structurally analogous circuits.

Our theory expands on existing work on action selection in the basal ganglia, and addresses the contributions of cortex to this process. As such, we adopt the terminology of an actor-critic reinforcement learning architecture (Sutton and Barto, 1998; O’Reilly and Frank, 2006) to describe the computational roles of basal ganglia and the dopamine system. The basal ganglia functions as an *Actor* that decides which action to take (or in our extended model, which plan to pursue). The dopamine release system, including amygdala, ventral striatum, and related areas, serves as a *Critic* by gauging the success of each action relative to expectations. Phasic dopamine release from this critic system serves as a reward prediction error learning signal for the basal ganglia actor system.

To this existing computational/biological theory we add two new computations, each made by participating regions of cortex. The first is a *Proposer* component. This system takes information about the current situation as input, and produces a single candidate plan representation. This proposer functional role may be less important for laboratory tasks, since they usually have a small set of actions (e.g., levers, yes/no responses), which can be learned thoroughly enough to process all options in parallel routes through the basal ganglia (e.g., Collins and Frank, 2014). However, dealing with unique real-world situations requires coming up with a potential approach before evaluating outcomes (e.g., different plausible routes in a trip planning context). This proposer system could use computations characterized as model-free, stimulus-response, constraint satisfaction, or model-based, depending on the complexity of the situation.

The other cortical addition is a *Predictor* component, which predicts the likely outcome of each proposed plan. In our model as currently implemented, this prediction always took place in two steps: predicting an “Outcome,” and from that outcome, predicting a “Result” or potential reward. We think that this type of prediction is actually performed by a variety of brain systems, using a variable number of steps for different types of decisions; but for the present purposes, it is adequate to simply think of this component as producing a prediction of an outcome by any means. This system’s computation is thus very much “model-based,” according to that terminology.

In our system, the Actor uses the predicted outcome (when available) of the proposed plan to either accept or reject that plan. Having this specific outcome prediction greatly simplifies the computational task of the actor component; it need simply accept plans that are predicted to have rewarding outcomes, and reject those that do not. If the proposed plan is rejected, the Proposer component makes a new, different proposal, a new prediction is made by the Predictor, and the Actor again decides to accept or reject that newly proposed plan. This operation proceeds serially until a candidate plan is selected. The serial, one-at-a-time plan consideration is slow, but computationally helpful in making an accurate prediction of outcomes in novel, poorly learned domains. It allows the full power of the cortex to be directed toward each prediction, and avoids binding problems, as we address further in the Discussion section.

This computational approach can attack complex problem spaces by sequentializing a complex decision into many sub-decisions, and allowing the actor component to accept or reject each proposed sub-plan or sub-conclusion. We propose that our ability to sequentialize a problem into sub-steps and make a binary decision for each is the source of humans’ remarkable cognitive abilities relative to other animals. This method of simplification may, however, have particular inherent weaknesses that explain some of humans’ notable cognitive biases.

### Continuum of Controlled/Goal-Directed vs. Automatic/Habitual

Due to its sequential, hierarchical and multi-component nature, the model provides a mechanistic basis for a continuum of controlled/goal-directed vs. automatic/habitual behavior. At every sequential step, there is the potential for an outer-loop decision about what overall strategy to employ, e.g., whether to engage in further prediction, or iterate to another proposed plan, etc. Within that outer loop, there are more specific decisions regarding what factors to focus on, such as which branches to pursue in prediction, etc.

In cases of high urgency or low stakes, all of that complexity could be elided in favor of a quick thumbs-up (Go gating decision) from the Actor to the Proposer’s initial suggestion. This optimization for speed could be created by reinforcement learning in the basal ganglia, with inputs that capture timing and relevant time pressures. We suggest that this may represent the majority of habitual or automatic responding – a fast path through the very same circuits, typically at the lower levels of the abstraction hierarchy (e.g., involving supplementary motor areas and the dorsolateral striatum). Thus, consistent with the continuum perspective, and a surprising difficulty in finding explicit claims and data about what neural substrates uniquely support habitual behavior (e.g., Wickens et al., 2007; Seger and Spiering, 2011), there may be no separate neural substrate associated with habitual behavior – it is just the simplest and fastest mode of processing through the entire decision-making apparatus.

If this is the case, then it would seem to challenge the various attempts to establish strong dichotomies between e.g., model-free vs. model-based, or even value-based vs. value-free or belief-based vs. belief-free (Miller et al., 2018, 2019). In short, even habitual behavior depends on a (usually implicit) decision to not engage in a more controlled form of behavior, and that decision likely depends on assessments of the relevant “stakes” (values or utilities) in the current context, and the estimated cost/benefit tradeoffs in engaging in more effortful levels of control (Pezzulo et al., 2013).

Thus, estimated value is always in play, even in the context of habitual behavior. To reconcile this idea with the finding that habitual behaviors are relatively insensitive to changes in reward, we would need to determine the relative cost/benefit tradeoff estimates associated with the alternative options that might have been taken instead of performing the habitual response. Certainly, if the habitual response would lead to imminent severe harm, and this was obvious to the individual, then we would expect them not to engage in it. Typically when clearly erroneous habitual responding occurs in the real world, it can be traced to a lack of attention being paid to the relevant factors, likely resulting from prior decisions to allocate that attention elsewhere. In other words, taken literally, a purely habitual response presumes that the person is otherwise somewhat of a zombie. Instead, we suggest, consistent with others (e.g., Cushman and Morris, 2015) that habitual responses occur within a broader context (i.e., the outer-loop) of at least some level of cognitive control.

### The Model-Free Actor in the Loop

A central feature of our model is that the basal ganglia Actor system provides a value-based final Go/NoGo decision, even (and perhaps especially) under controlled, deliberative situations. The Actor fits the classic description of a model-free reinforcement learning system, and thus our framework says that there is an important model-free component to even high-level goal-driven and controlled behavior. This is consistent with a similar claim in the hierarchical model of Cushman and Morris (2015) and with their more recent experimental results (Cushman et al., this issue). Thus, whether one wants to call this Actor “model-free” or not, even when it receives all manner of highly-processed goal, internal state, and prediction inputs from the cortex, further challenges the utility of this terminology. Furthermore, as we noted above, the availability of predicted outcome representations from the Predictor component can make the Actor’s job very simple, and yet likely much more effective than a typical model-free system.

### The Central Role of the Proposer

The function of the Proposer is particularly central to our overall framework, as it serves as the starting point for any action/plan initiation process. As noted, we think it functions through parallel, constraint-satisfaction processing to integrate a large number of different constraints, cues, and other contextual information to arrive at a plausible plan of action in a given situation (O’Reilly et al., 2014). It is precisely through this dynamic integration process that otherwise subtle, unconscious factors may be able to have measurable influences over our behavior (Bargh, 2006; Huang and Bargh, 2014). In addition, this property of the proposer enables even habit-based behavior to be somewhat flexible and capable of incorporating novel constraints from the current environmental state – even habitual actions are not purely ballistic and “robotic” in nature (Cushman and Morris, 2015; Hardwick et al., 2019).

Furthermore, as we’ll see next, the incremental shaping of these Proposer representations over the course of learning plays a critical role in the automatization and habitization of behavior. Indeed, as the Proposer gets better and better at generating effective plans for increasingly well-known contexts, the Actor learns to essentially rubber-stamp these plans, thus resulting in fast, efficient habitual behavior. This happens through reinforcement learning shaping the weights from cortex to the basal ganglia Actor system; as the Actor sees more positive, rewarding examples, it becomes more biased toward a Go response. Along with its importance in habitual behavior, the Proposer component is also essential for coming up with plans in novel, challenging situations requiring controlled processing. Thus we argue that these functional distinctions may not have clear corresponding anatomical distinctions: the basal ganglia, Actor component is involved in all types of decisions, and that different areas of cortex may be recruited to play roles as Proposer, Predictor, and even to add more highly-processed inputs to model-free value predictions (Herd et al., 2019).

### Transition From Slow and Controlled to Fast and Automatic Processing

**FIGURE 3:**
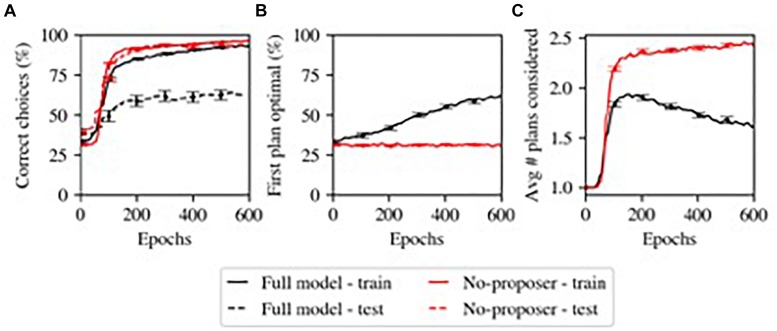
Model’s simulation of habitization (from Herd et al., 2019). Performance grows faster with more training, but generalization is sacrificed. **(A)** Performance (% correct). The model with the Proposer component (Full model) performs worse at generalization (test – dashed lines). **(B)** The Proposer component learns correct behavior over time, with increasing probability of producing optimal plans for first consideration. **(C)** The Proposer’s learning reduces the total number of plans considered, by providing good options for first consideration, and thus also reduces total performance time. This may capture one factor in habitization in humans and animals.

One of the main results from our computational model (Herd et al., 2019) is shown in Figure 3, where the Proposer component gradually learned to choose a Plan appropriate for the current situation and goal. Initially, without relevant domain knowledge, the Proposer generates plans essentially at random, and a larger number of iterations are required to arrive at a Plan that the Actor approves of. Over the course of learning, the more appropriate initial plans generated by the Proposer reduces the number of iterations required, and thus the overall model gradually transitions from a more serial, iterative mode of processing to a more parallel mode of processing dominated by the parallel constraint-satisfaction dynamic in generating plans in the Proposer system. This illustrates a continuum of habitization occurring over learning within the same overall system. Furthermore, the Proposer was able to learn only when the remaining systems chose to pursue a given plan; its learning was thus guided by the other systems, including the Predictor component.

Our initial model does not include the outer-loop ability to select which decision-making processes to engage in, so it did not have the ability to further optimize decision making by not engaging the Predictor at all, which would have resulted in even greater speedup, and corresponds with a more purely habitual response mode. We are currently working on a version of the model with this functionality.

### Goal-Directed Behavior From a Model-Free System

In our model, the input to the Proposer system includes information about goals, so the behavior produced by this system qualifies as goal-directed, despite the relatively simple computations. Most computational work on model-free reinforcement learning systems addresses systems that do not include current goals as inputs. Those systems can only produce habitual behavior. However, there does not appear to be any strong justification for this assumption, and it seems more reasonable (as well as empirically justified) to assume that the relevant systems in the mammalian brain have access to a variety of useful information, including current goals. Indeed, there has been some discussion of goal-directed habits in other literature (Verplanken and Aarts, 1999; Aarts and Dijksterhuis, 2000).

When we assessed the accuracy with which the Proposer component produced a Plan which accomplished the current Goal (with Situation and Goal chosen at random from ten and four possibilities, respectively), we observed that this component displayed goal-directed behavior by matching Plans to Goals at an above-chance level, but learned slowly (Figure 3). This matches the slow transition from controlled to automatic processing (Gray et al., 1997) (note that we did not optimize parameters for Proposer learning in this task; some other parameterizations did produce better and faster learning).

Thus, our model illustrates one case in which goal-directed behavior results from thoroughly model-free computations.

### Serial Processing Enables Coherent Predictions

A key advantage of the serial evaluation of different proposed plans in our model is that it allows many different brain areas to contribute to the evaluation process, without suffering from the *binding problem* that would otherwise arise from an attempt to evaluate multiple options in parallel. For example, if two options are considered together, and another brain area generates an activation associated with a prediction of difficulty, while another activates a prediction of relative ease, how do we know which prediction goes with which option?

This is analogous to the binding problem in visual search, where serial processing has also been implicated as a solution (Treisman and Gelade, 1980; Wolfe, 2003). For example, people cannot identify in parallel whether a display contains a particular *conjunction* of features (e.g., a red X among green Xs and red Os), whereas they can identify separable features in parallel (just Xs among Os, or just red among green). Likewise, the conjunction of options and their predicted consequences at many different levels in the brain, which likely depends on the current internal and external state, can be much more coherently evaluated by considering options one at a time. Furthermore, this serialization of the processing enables the *same* predictive and evaluative neural representations to be re-used across different situations and contexts, thus facilitating the transfer of knowledge to novel situations. In short, more complex model-based, predictive forms of control must involve serial processing mechanisms.

However, there are costs associated with serial processing, not only in terms of time, but also in terms of the coordination and control required to organize the serial processing itself. In addition, evaluating any one option relative to the predicted properties of other options requires some form of maintenance and comparison operations across these predictions, placing demands on working memory and other limited cognitive resources. Nevertheless, there are strong serial-order effects on decision-making, which such a serial model can naturally account for, so future modeling work will need to address these challenges in order to better address the complexities of these phenomena.

In summary, our sequential, integrated, systems-based approach provides some potentially novel perspectives on central questions about the nature of controlled/goal-driven vs. automatic/habitual behavior.

## Discussion

We have presented a computational systems-neuroscience approach to understanding the dynamics of decision making and action selection, which suggests that the classical dichotomy between habitual/automatic vs. goal-directed/controlled processing can be understood as different modes of functioning within a unitary system, operating fundamentally in a serial manner. The serial nature of the processing affords a natural incrementality to the continuum between these modes of processing – as the system iterates longer and engages more elaborated predictive and evaluative forms of processing, it shades more toward the goal-driven, controlled-processing end of the spectrum. By contrast, there is a fast track through the system where a proposed plan of action is derived rapidly through parallel constraint-satisfaction processing, which is then quickly approved by the basal ganglia Go/NoGo system – this corresponds to the habitual end of the spectrum. However, even this habitual level of behavior is contingent on an outer-loop of decision making that has established relevant thresholds and control parameters to enable the fast-track to be taken in the first place. Thus, habitual behavior still operates within an at-least minimally controlled context, in situations where the overall benefits of so behaving make sense compared to investing greater levels of control.

This framework contrasts with the dual-pathway model proposed by Miller et al. (2019) and similar models which suggest that habitual and controlled, goal-driven processing are subserved by parallel pathways that compete via an Arbiter system for control over behavior. It also contrasts with other models having a similar overall structure, but which use model-free and model-based components that likewise require an Arbiter system (e.g., Daw and Dayan, 2014). The framing of the interrelationship of habitual and controlled processing provided by Cushman and Morris (2015) is much more consistent with our framework, but further work is required to establish more detailed comparisons between their implemented models and our model. Likewise, the Pezzulo et al. (2013) model shares the central idea that model-based predictive mechanisms are only engaged when they yield additional value, and we will be working to relate their computational-level mechanisms to the more biologically based framework we have developed.

Behaviorally, there are several important predictions that our model makes, which can be tested empirically. For example, consistent with a great deal of theory as well as folk psychology, we argue that habitual control is only enabled in either low-stakes or highly urgent situations. How does this outer loop of control interact with the various behavioral paradigms that have established the relative value-insensitivity of habitual behavior (Wood and Rünger, 2016)? Can our model account for both this value-insensitivity but also the cases where relevant expected reward values shift the system to more controlled, goal-driven behavior? What behavioral paradigms can effectively test such dynamics? One recent result provides a nice confirmation of one of our model’s core predictions: that habitization is primarily about rapid activation of a good proposed plan of action (i.e., the Proposer in our model), but there remains a final “goal-directed” process (the Actor in our model) responsible for actual action initiation (Hardwick et al., 2019).

Another fertile ground for testing the model is in the domain of serial order effects on decision-making. For example, the balloon analog risk task (Lejuez et al., 2003; White et al., 2008; Van Ravenzwaaij et al., 2011; Fukunaga et al., 2012; Fairley et al., 2019) involves making a long sequence of decisions about whether to keep pumping a simulated balloon, or cash out with a potentially sub-optimal level of reward, and it seems uniquely capable of capturing real-world individual differences in propensity toward risky behaviors (Lejuez et al., 2003; White et al., 2008). Various sources of evidence suggest that there is something about the sequential nature of this task that is critical for its real-world validity. Thus, we are actively exploring this question in terms of the serial processing present in our model. In addition, there are other well-established serial-order effects in decision making, including framing effects (Tversky and Kahneman, 1981; De Martino et al., 2006), and the widely-studied foot-in-the-door and door-in-the-face strategies (Pascual and Guéguen, 2005), which our serial model is particularly well-suited to explain.

## Author Contributions

All authors contributed to the development of the ideas and computational models described herein, and to the writing of the manuscript.

## Conflict of Interest

RO’R, AN, and SH were employed by company eCortex, Inc. The remaining author declares that the research was conducted in the absence of any commercial or financial relationships that could be construed as a potential conflict of interest.
